# Socioeconomic Differences in Vaccination Coverage After a Mandatory Vaccination Law, 1855-1900

**DOI:** 10.1001/jamanetworkopen.2024.60558

**Published:** 2025-02-19

**Authors:** Susanna Ukonaho, Virpi Lummaa, Michael Briga

**Affiliations:** 1 Department of Biology, University of Turku, Turku, Finland; 2 PandemiX Centre of Excellence, Roskilde University, Roskilde, Denmark; 3 Charité Centre for Global Health, Charité Universitaetsmedizin, Berlin, Germany

## Abstract

**Importance:**

Mandatory vaccination is a major tool to combat increasing vaccine hesitancy. In principle, a vaccination law, ie, a mandatory vaccination law without exemptions, applies equally to everyone, but its effects across different socioeconomic groups (SEGs) remain unknown.

**Objective:**

To examine the association of a vaccination law with vaccination coverage in different SEGs during 1855 to 1900.

**Design, Setting, and Participants:**

This population-based cohort study monitored 45 years (1855-1900) of Finland’s first vaccination campaign against smallpox to estimate the association of the 1883 vaccination law with vaccination coverage in infants (age <1 year) across different SEGs. Data were analyzed from October 2023 to January 2024.

**Exposure:**

A mandatory smallpox vaccination law for all children.

**Main Outcomes and Measures:**

Vaccination status was determined from vaccination records and defined as receiving 1 dose of the smallpox vaccine. The primary outcome was the annual vaccination coverage in different SEGs and its change before vs after the vaccination law.

**Results:**

A total of 40 008 children aged less than 1 year were included. The high SEG had high vaccination coverage, at a mean (SD) of 90% (49 percentage points), and the law was associated with halting its declining trend. For the middle SEG, the law was associated with a 26-percentage point increase in coverage, to a mean (SD) of 83% (50 percentage points). For the low SEG, the law had no association with vaccination coverage, which always remained below 35% (mean [SD]: prelaw, 26% [22 percentage points]; postlaw, 32% [23 percentage points]).

**Conclusions and Relevance:**

In this cohort study, a historic vaccination law was not associated with increased vaccination in the SEG with the lowest vaccination coverage, emphasizing the need for additional interventions to increase vaccine uptake in low-coverage communities.

## Key Points

**Question:**

Are vaccination laws associated with decreased socioeconomic disparities in vaccination coverage?

**Findings:**

In this 45-year cohort study of 40 008 children younger than 1 year in Finland from 1855 to 1900, an 1883 mandatory vaccination law was associated with increasing vaccination coverage in the middle socioeconomic group by 26 percentage points, to 86%, but had no significant association in the low socioeconomic group, whose coverage remained below 35%.

**Meaning:**

These findings suggest that even successful vaccination laws can fail to address the socioeconomic disparities in vaccination coverage.

## Teaser

This population-based cohort study examines the association of an 1883 mandatory vaccination law with vaccine coverage rates among infants aged less than 1 year in Finland from 1855 to 1900.

## Introduction

Vaccines represent a major intervention against infectious diseases. For example, the rollout of childhood vaccines is associated with historic reductions in the incidence of childhood infections and childhood mortality.^1,2,3^ Yet, in high-income countries, declining vaccination coverage has triggered a resurgence of vaccine-preventable infections, such as measles,^4,5,6,7,8^ leaving infants unprotected and failing the regional World Health Organization elimination goals for infections such as measles and rubella.^9^

The World Health Organization declared vaccine hesitancy, ie, the delay in acceptance or refusal of vaccination despite the availability of vaccination services,^10^ 1 of the top 10 global health threats.^11^ Decreases in vaccination coverage, together with the resurgence of many vaccine-preventable childhood infections, have incentivized public health authorities to introduce a form of mandatory vaccination. For example, starting from 2017 onwards, in Italy,^12^ Germany,^13^ France,^14^ and several US states (eg, California, Illinois, and Connecticut^15,16,17^), children can access public schools only after vaccination against several childhood infections, including measles, mumps, and rubella (MMR vaccine); varicella; and diphtheria, tetanus, and pertussis (DTaP vaccine).

While several studies have shown that vaccination laws, exemptions aside, are effective at increasing vaccination coverage,^12,14,17^ the effect of laws across different socioeconomic groups (SEGs) remains unknown, even in a post–COVID-19 era.^18,19^ On the one hand, if a vaccination law is equally effective for all, it could equalize the coverage between various communities or SEGs. Alternatively, communities with low vaccination coverage can face more barriers to immunization, including reduced access to vaccines, higher vaccine hesitancy, and possibly a stronger antivaccine response following the implementation of vaccine mandates.^19,20,21,22,23^ Such differences are problematic because they could have the unintended consequence of neglecting or even reinforcing socioeconomic or community differences in vaccination coverage and infectious disease incidence.

In this study, we test the association of the first law requiring vaccination against childhood smallpox infection with infant vaccination coverage across SEGs in 19th century Finland. Smallpox was a highly lethal childhood infection until the development of the world’s first vaccine by Edward Jenner in 1798.^24^ In Finland, smallpox vaccination was introduced in 1802.^25^ Vaccination campaigns were carried out annually in summer in most parishes^26,27^ (eTable 1 in Supplement 1). For many decades, vaccination was voluntary, but infant vaccination coverage remained below 70% until the first vaccination law in 1883,^25^ a mandatory law without exemptions, increased the coverage of infants aged less than 1 year by 20 percentage points.^26^ The vaccination law was enforced through a monetary fine.^25^ Following historical descriptions, this fine was implemented gradually over time, but the percentage of vaccine refusers receiving a fine and the amount of the fine remains unclear.^25^ Introducing the smallpox vaccine profoundly reduced the incidence of smallpox epidemics, which decreased from every 4 years in the prevaccine era to every 8 years in the vaccine era.^2^ However, regular smallpox outbreaks remained and caused significant mortality until and, to a smaller extent, even after the 1883 vaccination law was adopted (eFigure 1 in Supplement 1). Here, we monitor infant vaccination coverage for 45 years (1855-1900) across SEGs in Finland and test whether the 1883 law was associated with decreased or exacerbated socioeconomic differences in coverage.

## Methods

This cohort study was conducted according to Finnish law, which states that studies using historical data more than 100 years old does not require permits from an ethics committee or informed consent. This study is reported following the Materials Design Analysis Reporting (MDAR) Framework for transparent reporting in the life sciences.

### Data Source

In 18th and 19th century Finland, the Lutheran Church was obligated by law to maintain population census records of all births, deaths, marriages, migration between parishes, and other details of parish members. These original records are stored in the National Archives of Finland. In this study, we used birth, death, vaccination, and socioeconomic records from church books of 10 parishes across Finland (Ikaalinen, Karvia, Kustavi, Parkano, Jämijärvi, Honkajoki, Kuopio, Kuopio countryside, Tuusniemi, and Maaninka), which we obtained following the local laws. Four parishes are located in center-eastern Finland (Kuopio region) and 6 in southwest Finland (Ikaalinen region) (eFigure 2 in Supplement 1).

### Vaccination Data

We photographed and digitized 2000 pages of vaccination records from the National Archives of Finland. In total, we collected 40 008 individual vaccination records from 1855 until 1900 that contained information on vaccinated children, including address, parents’ occupation, age, and date of vaccination. Our study focuses on the middle to late 19th century, as there are no consistent vaccination records with socioeconomic data from before the 1850s, and after 1900, smallpox mortality was negligible in Finland (eFigure 1 in Supplement 1).

### Socioeconomic Data

We used individual-level information on parental occupation to identify a child’s SEG. Because the social status of married women and their children was tied to that of the husband, we used men’s occupation as a reference for SEG. Following our previous study,^28^ we identified and translated 89 occupations from the vaccination records, which we classified into 3 SEGs: high, which includes all those who belonged to the 4 estates (ie, nobility, clergy, burghers, and landed farmers); middle, including sharecroppers and craftsmen; and low, including servants, dependent lodgers, and vagabonds. Nationwide percentages of the main occupations are provided in eTable 2 in Supplement 1. For the 10 parishes in our study, a mean of 16% of men belonged to the high SEG, 26% to the middle SEG, and 58% to the low SEG, and these values were consistent across censuses (1860: high, 15%; middle, 28%; low, 57%; 1880: high, 19%; middle, 22%; low, 59%; 1890: high, 14%; middle, 29%; low, 58%).

### Estimation of Vaccination Coverage

We calculated vaccination coverage following our previous study^26^ applied to data per SEG (eFigure 3 and eTable 3 in Supplement 1). The numerator of the vaccination coverage is the annual number of vaccinated individuals aged less than 1 year per parish per SEG obtained from the historical vaccination records. The denominator is the estimated annual number of births per parish per SEG, subtracting the number of deaths before age 1 year. Note that in 19th century Finland, infant mortality rates were high, ranging between 20% and 30%.^29^ To identify the number of births and deaths per SEG, we used the data from 3 occupational censuses of men, ie, all males older than 15 years from the years 1860, 1880, and 1890.

We performed 2 sensitivity analyses to our estimates of vaccination coverage. First, a previous study on this population found that lower SEGs had lower birth rates and higher infant mortality rates.^28^ Because the men’s occupational data do not account for these socioeconomic differences, our approach could create an overestimation of vaccination coverage of the high SEG and an underestimation for the middle and low SEGs. Hence, we repeated all analyses using the women’s and children’s 1880 occupational census data as the denominator. Because these data include all children until age 15 years, they correct for socioeconomic differences in birth and child mortality rates until age 15 years. Both approaches, ie, using 1860, 1880, and 1890 men’s occupational status and the 1880 women’s and children’s occupational data, gave consistent results (eFigure 4 in Supplement 1). In this study, we present the analyses using the men’s occupational data, as these are based on 3 censuses. Second, in 19th century Europe, many teenaged individuals would transiently work as servants, a low SEG profession, before starting a family.^30^ Because these individuals rarely reproduce at this age, including them can underestimate the vaccination coverage in the low SEG. Hence, we also estimated the vaccination coverage in the low SEG excluding all servants from the data, and the conclusions were consistent with our main findings (eFigure 5 and eTable 4 in Supplement 1).

### Statistical Analysis

We performed all statistical analyses using R software version 3.6.1 (R Project for Statistical Computing). We investigated whether the 1883 vaccination law was associated with abrupt changes in vaccination coverage between SEGs using threshold models fitted as described in detail in our previous studies.^26,31^ Threshold models allow identifying the year with major changes in vaccination coverage using model fitting criteria (eFigure 6 in Supplement 1), thereby avoiding the arbitrary comparison of the eras before vs after the vaccination law. Generalized additive mixed models provide an alternative smoothened approach to analyze changes in vaccination coverage over time.^32^ In brief, these analyses gave results that were consistent with those obtained with the threshold models (eAppendix, eFigure 7, and eTable 5 in Supplement 1).

We fitted threshold models in R using the function glmmTMB of the package glmmTMB^32^ and identified the best-fitting model based on the second-order Akaike Information Criterion (AICc) using the function dredge from the package MuMIn.^33^ Better-fitting models have lower AICc and models within 4-unit difference in AICc are considered plausible.^34^ To estimate the confidence around the threshold, we used a 4-unit difference in AICc confidence interval (4-unit AICc CI), ie, we identified the threshold years that fitted within 4 units of the AICc of the best-fitting threshold. To confirm that the threshold model was the best fit, we compared the model fit of linear models with and without threshold. We also compared model fits using Akaike weights,^35^ which represent the likelihood of a model relative to the tested models with a value ranging from 0 (most unlikely) to 1 (most likely).

To account for the variation in vaccination coverage between parishes, all models contained parish identity as a random intercept, and we accounted for temporal autocorrelation by including corAR1 autoregression structure.^36^ Including parish identity and temporal autocorrelation improved the model fit (eg, difference in AICc = −62). The final models fulfilled all assumptions, checked with the function simulateResiduals of the package DHARMa^37^ and without temporal autocorrelation (autocorrelation function < 0.1).

*P* values were 2-tailed, and statistical significance was set at *P* ≤ .05. Data were analyzed from October 2023 to January 2024.

## Results

The cohort included 40 008 children aged less than 1 year from 1855 to 1900, including 37 years (1855-1882) and 20 695 vaccination records (52%) before the 1883 vaccination law and 17 years (1883-1900) and 19 313 vaccination records (48%) after the law (Table 1; eTable 1 in Supplement 1). Stratified by SEG, there were 13 809 individuals from the high SEG, 12 717 individuals from the middle SEG, and 13 482 individuals from the low SEGs (Table 1).

**Table 1.** Vaccination Coverage Among Infants Younger Than 1 Year Per Socioeconomic Group Before (1855-1882) vs After (1883-1900) the Start of the 1883 Mandatory Vaccination Law

Socioeconomic group	No. vaccinated		Vaccination coverage, mean (SD), % (percentage points)^a^	
	Prelaw period	Postlaw period	Prelaw period	Postlaw period
High	5273	8536	93 (62)	90 (49)
Middle	7898	4819	57 (53)	83 (50)
Low	7524	5958	26 (22)	32 (23)

^a^ Coverages are means for 10 parishes for all years per era.

### Changing Vaccination Coverage in the Early 1880s

**Figure 1.**
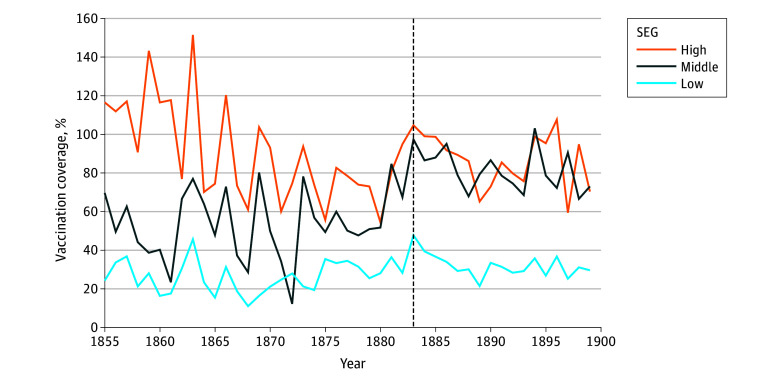
Mean Annual Vaccination Coverage Per Socioeconomic Group (SEG) for 10 Parishes in 19th Century Finland The vertical dashed line indicates the year 1883, when the mandatory vaccination law was introduced.

The mean (SD) population-level vaccination coverage for 40 008 children younger than 1 year was 59% (55 percentage points) before and 68% (49 percentage points) after the vaccination law (Figure 1 and Table 1). We tested whether there was an abrupt change in vaccination coverage using threshold models with data at the population level and per SEG. All models gave consistent results, with thresholds occurring in the interval between 1881 and 1883 (4-unit AICc CI, 1880-1890) (Table 2; eTable 3, eTable 4, eFigure 4, and eFigure 6 in Supplement 1), but the temporal trends in vaccination coverage differed between SEGs (interaction of SEG × threshold difference in AICc = −154) (Table 2). Hence, all SEGs showed changes in vaccination coverage when the vaccination law was introduced, but the changes were different among the SEGs.

**Table 2.** Statistical Analysis of the Time Series of the Number of Vaccinated Infants Aged Younger than 1 Year Relative to the Number of Births, Per Parish, Year, and SEG

Variable	AICc	Difference in AICc	Weight	Threshold^a^	Coefficient (year^−1^)
**Overall**					
Intercept	3443.15	0	0	NA	NA
SEG	3296.74	−146.41	0.02	NA	NA
Year	3439.76	−3.39	0	NA	NA
Year × SEG threshold interaction	3288.73^b^	−154.43^b^	0.98^b^	1882 (1880 to 1883)^b^	NA
**High SEG**					
Intercept	885.82	0	0.01	NA	NA
Year	887.27	1.45	0.01	NA	1.00
Year threshold interaction	877.18^b^	−8.64^b^	0.98^b^	1881 (1880 to 1882)^b^	Prethreshold: −2.23; postthreshold: −0.39
**Middle SEG**					
Intercept	1169.72	0	0.01	NA	NA
Year	1165.14	−4.58	0.14	NA	1.02
Year × SEG threshold interaction	1161.48^b^	−8.24^b^	0.85^b^	1882 (1880 to 1883)^b^	Prethreshold: −0.48; postthreshold: −0.51
**Low SEG**					
Intercept	1083.57	0	0	NA	NA
Year	1060.73	−22.84	0.15	NA	1.02
Year × SEG threshold interaction	1057.23^b^	−26.34^b^	0.85^b^	1881 (1880 to 1889)^b^	Prethreshold: 0.20; postthreshold: −0.26

Abbreviations: AICc, second-order Akaike information criterion; NA, not applicable; SEG, socioeconomic group.

^a^ Confidence interval defined as 4-unit difference in AICc.

^b^ Best-fitting model.

### Vaccination Coverages Before the Law

Before the law, the vaccination coverage varied more than 3-fold between SEGs: the high SEG had high coverage, with a mean (SD) of 93% (62 percentage points), while the middle and low SEGs had lower vaccination coverages, at 57% (53 percentage points) and 26% (22 percentage points), respectively (Table 1). These differences were highly statistically significant (difference in AICc = −154). Of note, the high and middle SEGs, but not the low SEG, showed declines in vaccination coverage over time (high: β = −2.2 percentage points per year; middle: β = −0.48 percentage points per year; low: β = 0.20 percentage points per year) (Table 2). Hence, before the law, higher SEGs had a higher vaccination coverage, but their coverage declined over time.

### Vaccination Coverages After the Law

**Figure 2.**
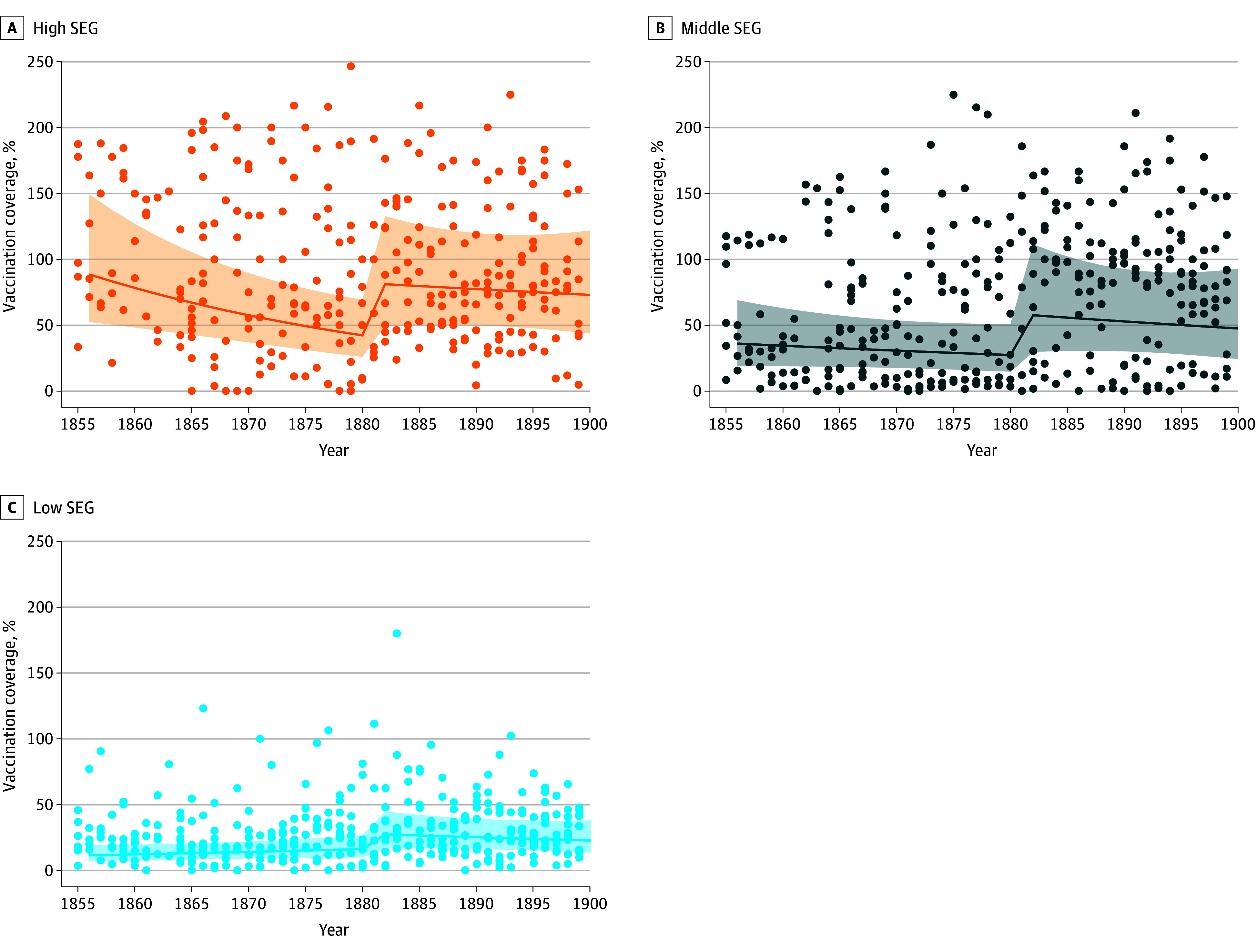
Vaccination Coverage With the Fit From Threshold Models Line indicates estimated curve; shading, CI; dots, individual data points. A, For the high socioeconomic group (SEG), the best-fitting threshold model shows the highest vaccination coverage with a declining trend over time and a threshold in 1881 (4-unit difference in second-order Akaike Information Criterion [AICc] CI, 1880-1882). B and C, The middle and low SEGs had lower vaccination coverages followed by an increase in the threshold years 1882 (4-unit AICc CI, 1880-1883) and 1881 (4-unit AICc CI, 1880-1889).

After 1883, the vaccination coverage of the high SEG remained at a mean (SD) of 90% (49 percentage points) (Table 1), but the threshold model showed that the prethreshold decline ended with the 1883 law. After the law, changes in coverage with time in the high SEG were not statistically significant (difference in AICc = −8.6; coefficient = −0.39) (Table 2 and Figure 2).

The threshold of the middle SEG had a 26–percentage point increase in vaccination coverage to a mean (SD) of 83% (50 percentage points) (difference in AICc = −8.2) after the law (Table 1 and Table 2). In contrast, the low SEG showed a minor increase in vaccination coverage by 6 percentage points, from a mean (SD) 26% (22 percentage points) to 32% (23 percentage points) (difference in AICc = −26.3) (Table 1 and Table 2). After the 1883 law, changes in coverage with time were small and not statistically significant (Table 2 and Figure 2). We repeated the analyses using generalized additive mixed models, which gave results that were consistent with the threshold models (eTable 5 and eFigure 7 in Supplement 1). Hence, for the SEGs with low vaccination coverage, the coverage increased after the law, but the increase for the low SEG was small compared with that of the middle SEG.

## Discussion

In this cohort study, we found that the first law requiring vaccination against smallpox—a mandatory law without exemptions—in a society with low vaccination coverage was associated with raising population-level vaccination coverage. However, the increase in vaccination coverage was largely driven by the middle SEG, for which a 26–percentage point increase in coverage persisted for at least 17 years. The low SEG showed only a 6–percentage point increase after the law, and their vaccination coverage remained at 32%. Vaccination laws are a public health intervention used to increase vaccination coverage, but to what extent their impact varies between SEGs and reaches communities with low coverage remains unknown.^18,19^ Our findings suggest that while vaccination laws can be effective at increasing vaccination coverage, their impact on decreasing socioeconomic disparities in vaccination coverage remains poor because they insufficiently reach the communities with the lowest coverage.

Our study found that infants from high SEGs had higher vaccination coverage in 19th century Finland. This is consistent with several studies in contemporary societies that found positive associations between parental income or education level and infant vaccination coverage, although there are differences among countries. For example, MMR vaccination coverage was higher in families with higher income or education in the UK, Ireland, the Netherlands, Greece, and Australia.^38,39,40,41,42^ In contrast, in Sweden and Germany, there was no association between parental income and/or education level and the coverage of MMR and other vaccines,^43,44^ and certain Italian provinces showed an opposite trend, namely that infants of parents with higher income or education had the lowest vaccination coverage.^45,46^ This variable association between socioeconomic status and vaccination coverage can have different causes. Some studies have suggested that lower SEGs can be more hesitant toward vaccines,^22,47,48,49^ but it is also possible that the high SEG experience a privilege paradox or prevention paradox,^50,51^ in which families that have better access to good quality health care have become indifferent toward vaccination, possibly mediated by limited exposure to infections. Hence, there is no consistent association between infant vaccination coverage and parent’s socioeconomic status in contemporary European Union countries.

The impact of mandatory vaccination on vaccination coverage can be variable and remains highly debated.^20,21,52^ In our study, mandatory vaccination was associated with increasing vaccination coverage by more than 20 percentage points. This increase is consistent with several studies in contemporary populations (France, Italy, and the US^12,14,17^) and supports the use of vaccination laws to combat declining vaccination coverages as currently observed for several routine childhood vaccinations in high-income countries.^5,6,7^ However, this increase was largely driven by the middle SEG, and the law was not associated with a meaningful improvement in coverage among the low SEG. In contemporary populations, we do not know the impact of vaccination laws on vaccination coverage across different socioeconomic communities. One study used regional socioeconomic differences in Australia and found that a “No Jab No Pay” policy, which eliminates nonmedical vaccination exemptions to receive government benefits, also was not associated with increasing vaccination coverage in low-coverage regions.^53^ Hence, it is possible that vaccination laws alone are ineffective in communities with the lowest vaccination coverage, indicating that further support, such as allowing time off from work or improved physical access to vaccination events, might be needed.^54^

### Limitations

Our study has some limitations. First, we defined SEGs based on parents’ occupation. This is not as robust as parental income, but in 19th century Finland, occupation correlated well with wealth.^55^ Second, there are parish-years with vaccination coverage estimates exceeding 100%. This is likely caused by population movement: during annual vaccination events,^26^ vaccines were given to families that visited from other parishes. Third, we cannot identify the causes for the positive association between SEGs and infant vaccination coverage: the low SEGs may have limited access to vaccines or higher vaccine hesitancy. Fourth, contextualizing to what extent our results apply to contemporary data remains challenging. There are several similarities in the dynamics of vaccination coverage between our study population and contemporary high-income societies, such as the positive association of vaccination laws with vaccination coverage^12,14,17^ and the positive association between socioeconomic status and vaccination coverage.^38,39,40,41,42^ However, the large socioeconomic differences in vaccination coverage and income in our study can create more pronounced results than in some contemporary high-income societies. Furthermore, the vaccination law analyzed here was enforced via a fine, which was implemented gradually after 1883.^25^ This differs from many contemporary vaccine mandates, in which vaccines are required to access public schooling.^12,13,14,15,17^ Such a mandate could not be implemented in 19th century Finland because, at that time, for most Finns, the schooling system consisted of short-term traveling schools, where an educator would provide teaching for a few weeks before going to another parish.^56^

## Conclusions

In this cohort study, we studied whether vaccination laws, exemptions put aside, could reach everyone equally and showed that such expectations were not realized. Public health authorities in historical and contemporary societies have struggled to implement vaccination laws, which require investments and organizational support. Our findings support the adoption of vaccination laws to increase vaccination coverage but also suggest that laws alone are likely insufficient to reach the communities with the lowest vaccination coverage. Our study indicates the need for additional interventions, such as information campaigns and easier access to vaccines,^20,54,57^ which can be specifically aimed at increasing vaccine uptake in communities with low vaccination coverage.

## Notes

Supplement 1. **eFigure 1. **Nationwide Smallpox Mortality in Finland, 1751-1940 **eFigure 2. **Map of the 10 Study Parishes **eTable 1. **Descriptive Statistics on the Vaccination Data Per Parish and SEG **eTable 2. **Distribution of the Socioeconomic Groups in Finland in 1865 Using National-Level Data **eFigure 3. **The Threshold Model of Vaccination Coverage in all 10 Study Parishes With Newly Added Data and 6/10 Overlapping Parishes Compared to the Previous Vaccination Coverage Model With 8 Parishes **eFigure 4. **Vaccination Coverage Estimates Using the Occupational Data of Women and Children Instead of Those of Working-Age Men **eFigure 5. **Vaccination Coverage Estimates Excluding Servants **eFigure 6. **AICc Threshold Profiles for Models in Table 2 **eTable 3. **Threshold Model Fit for a Model Without Socioeconomic Group, Using an Expanded Dataset **eTable 4. **Threshold Model Selection Table for Data Excluding Servants Based on the Second-Order Akaike Information Criterion **eAppendix.** Sensitivity Analyses: GAMMs **eFigure 7. **GAMM Fits and Their Derivatives for the Models in eTable 5. **eTable 5. **GAMM Model Selection Table for Models A-D Based on the Second-Order Akaike Information Criterion
eReferences.

Supplement 2.
Data Sharing Statement
