# Supplementary material for: Socioeconomic Differences in Vaccination Coverage After a Mandatory Vaccination Law, 1855-1900
Source: JAMA Netw Open. 2025 Feb 19;8(2):e2460558. doi: 10.1001/jamanetworkopen.2024.60558 (PMC11840648; doi:10.1001/jamanetworkopen.2024.60558)
Supplement: Supplement 1. — eFigure 1. Nationwide Smallpox Mortality in Finland, 1751-1940 eFigure 2. Map of the 10 Study Parishes eTable 1. Descriptive Statistics on the Vaccination Data Per Parish and SEG eTable 2. Distribution of the Socioeconomic Groups in Finland in 1865 Using National-Level Data eFigure 3. The Threshold Model of Vaccination Coverage in all 10 Study Parishes With Newly Added Data and 6/10 Overlapping Parishes Compared to the Previous Vaccination Coverage Model With 8 Parishes eFigure 4. Vaccination Coverage Estimates Using the Occupational Data of Women and Children Instead of Those of Working-Age Men eFigure 5. Vaccination Coverage Estimates Excluding Servants eFigure 6. AICc Threshold Profiles for Models in Table 2 eTable 3. Threshold Model Fit for a Model Without Socioeconomic Group, Using an Expanded Dataset eTable 4. Threshold Model Selection Table for Data Excluding Servants Based on the Second-Order Akaike Information Criterion eAppendix. Sensitivity Analyses: GAMMs eFigure 7. GAMM Fits and Their Derivatives for the Models in eTable 5. eTable 5. GAMM Model Selection Table for Models A-D Based on the Second-Order Akaike Information Criterion eReferences. [file jamanetwopen-e2460558-s001.pdf]

Supplemental Online Content

Ukonaho S, Lummaa V, Briga M. Socioeconomic differences in vaccination coverage after a mandatory vaccination law, 1855-1900. *JAMA Netw Open*. 2025;8(2):e2460558.
doi:10.1001/jamanetworkopen.2024.60558

eFigure 1. Nationwide Smallpox Mortality in Finland, 1751-1940

eFigure 2. Map of the 10 Study Parishes

eTable 1. Descriptive Statistics on the Vaccination Data Per Parish and SEG

eTable 2. Distribution of the Socioeconomic Groups in Finland in 1865 Using National-Level Data

eFigure 3. The Threshold Model of Vaccination Coverage in all 10 Study Parishes With Newly Added Data and 6/10 Overlapping Parishes Compared to the Previous Vaccination Coverage Model With 8 Parishes

eFigure 4. Vaccination Coverage Estimates Using the Occupational Data of Women and Children Instead of Those of Working-Age Men

eFigure 5. Vaccination Coverage Estimates Excluding Servants

eFigure 6. AICc Threshold Profiles for Models in Table 2

eTable 3. Threshold Model Fit for a Model Without Socioeconomic Group, Using an Expanded Dataset

eTable 4. Threshold Model Selection Table for Data Excluding Servants Based on the Second-Order Akaike Information Criterion

eAppendix. Sensitivity Analyses: GAMMs

eFigure 7. GAMM Fits and Their Derivatives for the Models in eTable 5.

eTable 5. GAMM Model Selection Table for Models A-D Based on the Second-Order Akaike Information Criterion

eReferences.

This supplemental material has been provided by the authors to give readers additional information about their work.

© 2025 Ukonaho S et al. *JAMA Network Open*.

Supplementary Information 1: Descriptive statistics and map

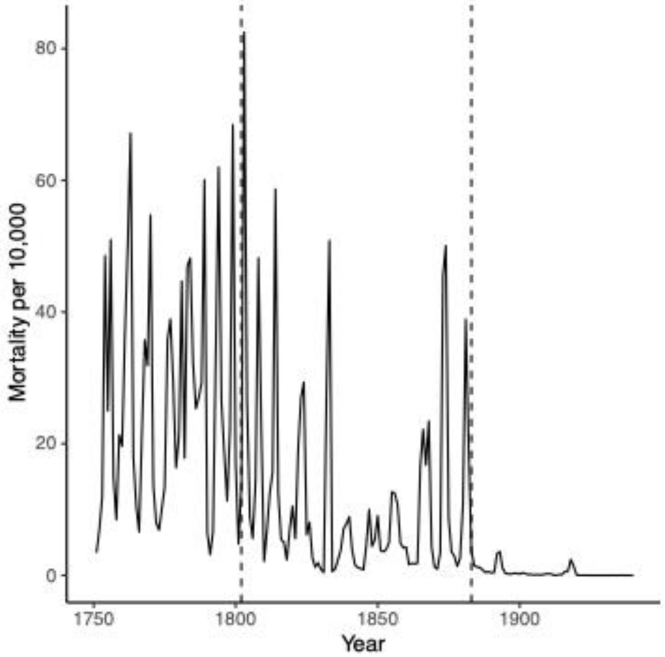

eFigure 1. Nationwide smallpox mortality in Finland 1751–1940. The vertical dashed line in 1802 shows the introduction of the smallpox vaccine, and, in 1883, the adoption of the vaccination law. Original data from Pitkänen, Mielke & Jorde 1989 (1).

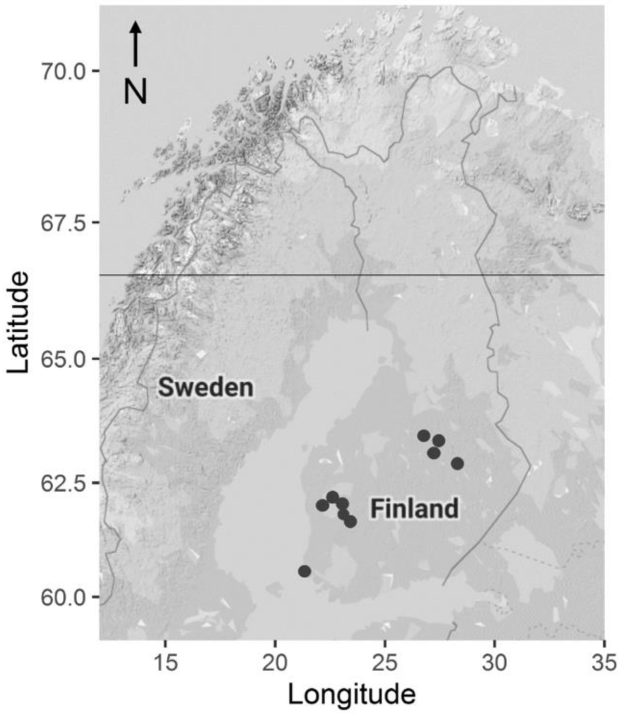

eFigure 2. A map of the 10 study parishes. The horizontal line indicates the Arctic Circle.

eTable 1. Descriptive statistics on the vaccination data per parish and SEG.

Parish	Parish size	Mean number of vaccinated annually	Mean vaccination month	Mean vaccination coverage age <1 (%)	Vaccination coverage per socio-economic group		
					High	Middle	Low
**Ikaalinen**	7700	164	June	67	94	87	20
**Jämijärvi**	2200	48	June	50	59	76	15
**Karvia**	2500	56	July	55	78	74	14
**Parkano**	3000	128	July	66	59	113	31
**Honkajoki**	2100	38	June	48	72	64	10
**Kustavi**	2200	43	June	79	113	116	43
**Kuopio city**	6800	76	June	36	28	18	62
**Kuopio countryside**	12000	284	June	40	123	13	34
**Maaninka**	4200	139	June	89	151	103	41
**Tuusniemi**	6000	116	June	68	139	57	23

eTable 2. Distribution of the socioeconomic groups in Finland in 1865 using national-level data from Rasila 2003 (2). Occupations of the high socioeconomic group are highlighted in light grey.

Occupation	%
Landowner farmer	38.4
Sharecropper	21.0
Farm worker	15.9
Unemployed	9.5
Servant	5.4
Landlord	4.0
Industry & retail	2.5
Nobility	2.0
Burghers	1.3

Supplementary Information 2: Sensitivity analyses 1– data selection and coverage estimation methods

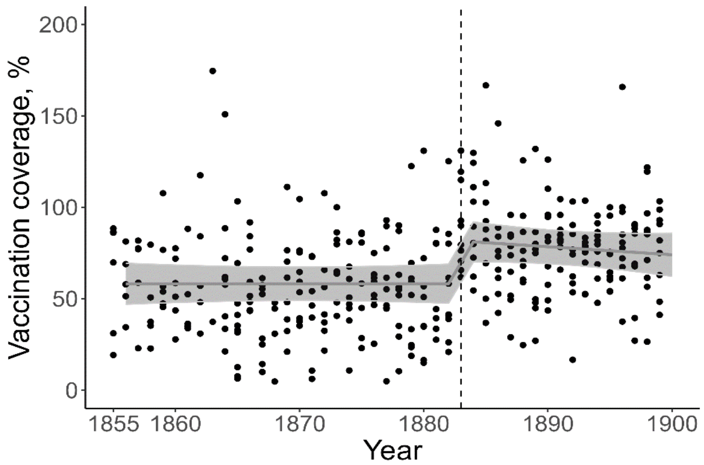

eFigure 3. The threshold model of vaccination coverage in all 10 study parishes with newly added data and 6/10 overlapping parishes compared to the previous vaccination coverage model with 8 parishes (3) gave consistent results. The predicted curve is shown in light grey line with the threshold of 1883 (indicated by the black dashed line), 95%CI in light grey band and data points per parish as black dots. Vaccination coverages were 13% and 22% lower than those described in our previous study (3), where the population-level coverages were 68% and 88% before versus after the law respectively. Vaccination coverage ranged from 0% to 198% and there were consistent parish-level differences in vaccination coverage albeit lower: 24% and 21% of the variance in vaccination coverage was explained by the random parish identity (eTable 3). Hence, the current study's 10% to 20% lower vaccination coverage is likely caused by the data covering different parishes.

© 2025 Ukonaho S et al. *JAMA Network Open*.

eTable 3. Threshold model fit for a model without socioeconomic group, using an expanded dataset relative to our previous study (3). In both studies, threshold models gave consistent results and indicated an increase in vaccination coverage starting from 1883 onwards. The model selection was done based on the second-order Akaike information criterion (AICc), with the AICc difference (ΔAICc) reflecting the absolute changes in model fits and the weights indicating the relative importance of the best-fitting model relative to the other tested models. The best-fitting model is shown in *italic*. 4ΔAICc shows the confidence interval around the threshold (see the methods section for an explanation on the 4ΔAICc 'confidence interval').

**Model**	**AICc**	**ΔAICc**	**weights**	**threshold**	**4ΔAICc**
**Threshold model without socio-economic groups**					
Intercept	3472.51	40.13	0		
Year	3445.53	13.16	0		
Threshold	*3431.04*	*0*	*1*	*1883*	*[1881,1883]*

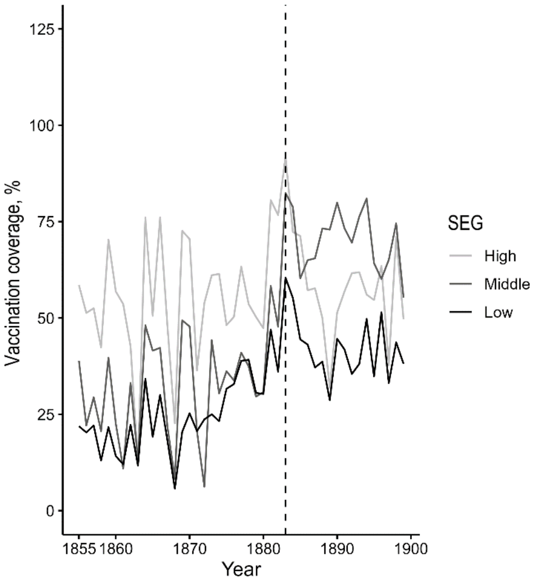

eFigure 4. Vaccination coverage estimates using the occupational data of women and children instead of those of working-age men shown in Figure 1. Note the consistent changes between the two approaches, with for example, the largest increase in vaccination coverage in the middle SEG. The vertical dashed line indicates the introduction of the vaccination law in 1883.

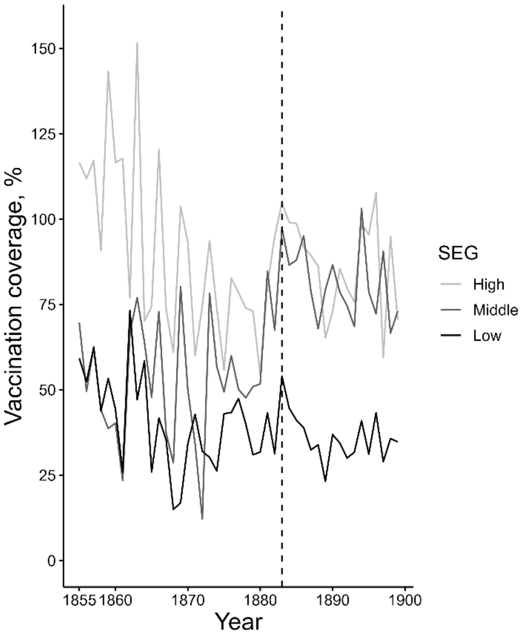

eFigure 5. Vaccination coverage estimates excluding servants show consistent results compared to the other two approaches (Figure 1 & eFigure 4). The vertical dashed line indicates the introduction of the vaccination law in 1883.

© 2025 Ukonaho S et al. *JAMA Network Open*.

eTable 4. Threshold model selection table for data excluding servants based on the second-order Akaike information criterion (AICc), with the AICc difference (ΔAICc), weights, threshold year, its 4ΔAICc confidence intervals and the transformed model coefficients (coeff) per era, which show annual percentage point changes in vaccination coverage. Best-fitting models are highlighted in *italic*. The full model (A) included all SEGs in one model, while model (B) is a subset of the low SEG.

Model	AICc	ΔAICc	weights	threshold	4ΔAICc CI	coeff ($year^{-1}$)
**A. Full model**						
**Intercept**	3441.00	0	0	NA	NA	NA
**SEG**	3351.11	-89.89	0.03	NA	NA	NA
**Year**	3440.19	-0.81	0	NA	NA	(see year models in B-D)
Year + SEG * Threshold	*3343.92*	*-97.08*	*0.97*	*1882*	*[1880, 1883]*	(see threshold models in B-D)
**B. Low SEG**						
**Intercept**	1148.75	0	0.23	NA	NA	NA
**Year**	1148.05	-0.70	0.33	NA	NA	1.02
Year * Threshold	*1147.48*	*-1.27*	*0.44*	*1889*	*[1880, 1890]*	*Pre-threshold: 1.02* *Post-threshold: 0.99*

Supplementary Information 3: Likelihood profiles for threshold models in Table 2

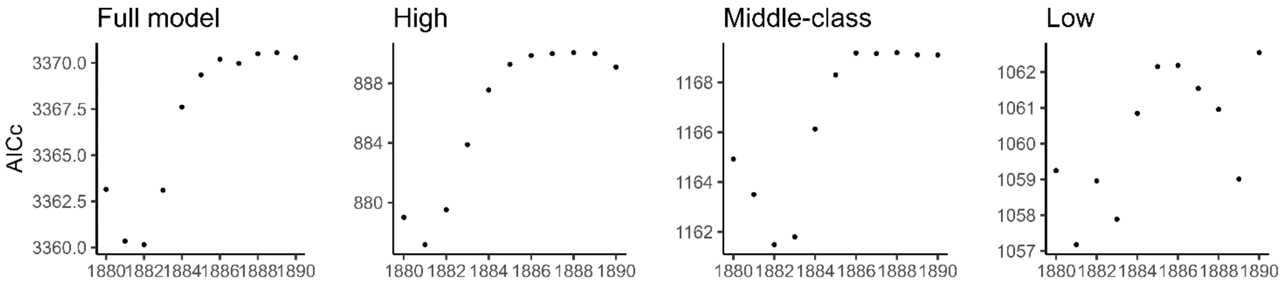

eFigure 6. AICc threshold profiles for models in Table 2 A-D. The figures show on the X-axis threshold at different years and on the Y-axis the fit of that model on the data. The dot with the lowest AICc is the best fitting model shown in Table 2 A-D and the 4ΔAICc 'confidence interval' is deduced from tested fits (see the methods section for an explanation on fitting threshold models and the associated 4ΔAICc 'confidence interval').

© 2025 Ukonaho S et al. *JAMA Network Open*.

Supplementary Information 4: Sensitivity analyses 2 – GAMMs

eAppendix. Sensitivity analyses – GAMMs

Methods: GAMMs analyses in R

General Additive Models (GAMMs) provide an alternative smoothened approach to quantify changes in vaccination coverage over time. Hence, we repeated the analyses from the threshold models using GAMMs with the function “gamm” of the package “mgcv” (4). We identified the years of change based on the inflection points of the GAMM derivatives with the function “fderiv” from the package “gratia” (5). In GAMMs, we noticed declining heteroscedasticity between SEGs, which we corrected by including weights on the variance per SEG (“varIdent”) in the function “weights” (6).

Results: Confirming the temporal trends of threshold models with GAMMs

GAMMs and their derivatives provide an alternative smoothened analysis of temporal changes in vaccination coverage per SEG. Consistent with the results of the threshold models, the GAMM models showed that changes in vaccination coverage differed between SEGs (model with year * SEG: ΔAICc = -193, eTable 5A). For the high SEG, GAMM derivatives showed a declining trend in vaccination coverage until the 1870s, followed by an increase, although GAMM derivatives overlapped with 0 and hence the changes were not statistically significant (eTable 5B, eFig. 7). In contrast, for both the middle and low SEGs, the vaccination coverage was low (eTable 5 C–D, eFig. 7), and GAMM derivatives showed an increase in vaccination coverage starting from the 1870s onwards, with the vaccination coverage reaching a maximum by the 1890s (eFig. 7, eTable 5C–D). Hence, GAMMs showed that the middle and the low SEGs reached the highest and most stable vaccination coverage just after the introduction of the vaccination law, and the middle SEG, but not the low SEG, showed the largest increase in the early 1880s, coinciding with the introduction of vaccination law.

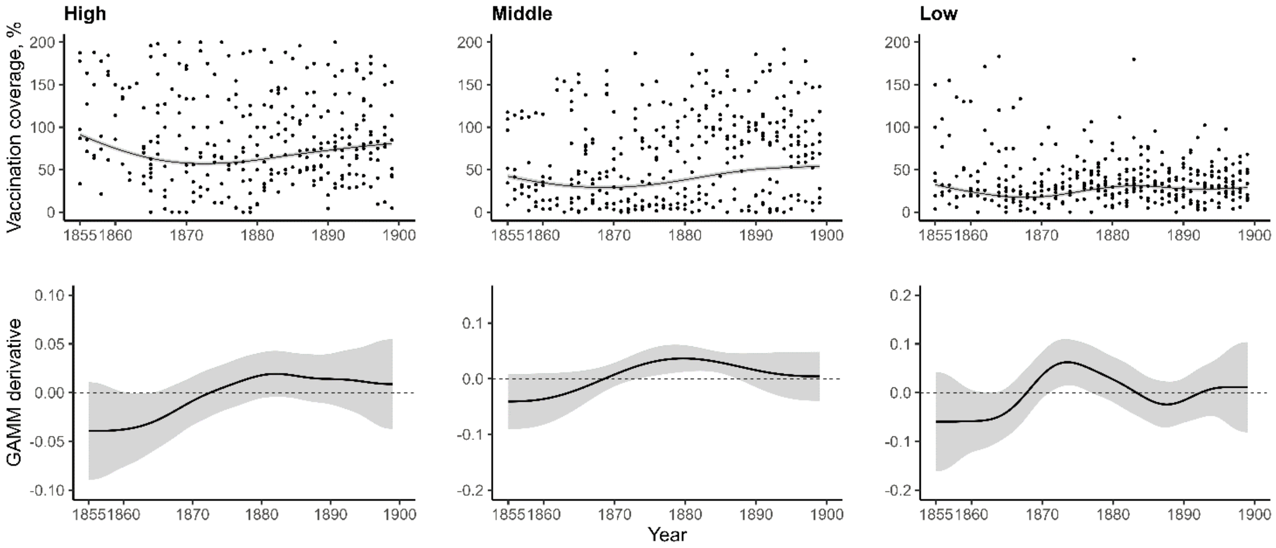

eFigure 7. GAMM fits (top) and their derivatives (bottom) for the models in eTable 5. In the high SEG, changes in vaccination coverage over time were not statistically significant. In the middle SEG, vaccination coverage improved starting from the 1860s and these became larger and statistically significant in the early 1880s (i.e., the higher derivative, the larger the increase in vaccination coverage). In the low SEG, there was an improvement in vaccination coverage starting from the late 1860s, which became statistically significant in the 1870s. For all SEGs, the vaccination coverage reached a plateau in the mid-1880s, after which there were no statistically significant changes. Vaccination coverages increase when the derivative is positive, i.e., above the dashed line showing 0.95% CI for the derivatives are shown using the grey band, with statistical significance inferred when the 95% CI do not overlap with 0.

© 2025 Ukonaho S et al. *JAMA Network Open*.

eTable 5. GAMM model selection table for models A-D based on the second-order Akaike information criterion (AICc), with the AICc difference (ΔAICc) and weights. The best-fitting model is shown in *italic*.

**Model**	**AICc**	**ΔAICc**	**weights**
**A. Full model**			
**Intercept**	3533.45	0	0
**SEG**	3354.90	-178.55	0
**Year**	3513.02	-20.43	0
SEG * Year	*3340.25*	*-193.20*	*1*
**B. High SEG**			
Intercept	*887.87*	*0*	*0.72*
**Year**	889.73	1.86	0.28
**C. Middle SEG**			
**Intercept**	1171.77	0	0.22
Year	*1169.28*	*-2.49*	*0.78*
**D. Low SEG**			
**Intercept**	1085.62	0	0
Year	*1060.12*	*-25.50*	*1*

© 2025 Ukonaho S et al. *JAMA Network Open*.

Supplementary Information 5: References

(1) Pitkänen KJ, Mielke JH, Jorde LB. (1989) Smallpox and its eradication in Finland: implications for disease control. *Population studies.* 43(1):95-111. doi:10.1080/0032472031000143866

(2) Rasila V. Suomen maatalouden historia. 1: Suomalainen yhteiskunta 1865. SKS Kirjat, Finland; 2003.

(3) Ukonaho, S., Lummaa, V., & Briga, M. (2022). The Long-Term Success of Mandatory Vaccination Laws After Implementing the First Vaccination Campaign in 19th Century Rural Finland. American journal of epidemiology, 191(7): 1180–1189. https://doi.org/10.1093/aje/kwac048

(4) Hartig, F. DHARMa: residual diagnostics for hierarchical (multi-level/mixed) regression models. 2020. https://cran.r-project.org/package=DHARMa. Accessed May 12, 2020

(5) Simpson, G.L. (2019) gratia: Graceful 'ggplot'-based graphics and other functions for GAMs fitted using "mgcv". https://cran.r-project.org/package=gratia

(6) Zuur, A.F., Ieno, E.N., Walker, N., Saveliev, A.A., Smith, G.M. (2009) Mixed effects models and extensions in ecology with R. New York, NY: Springer-Verlag.

© 2025 Ukonaho S et al. *JAMA Network Open*.
