# Supplementary material for: Socioeconomic Differences in Vaccination Coverage After a Mandatory Vaccination Law, 1855-1900
Source: JAMA Netw Open. 2025 Feb 19;8(2):e2460558. doi: 10.1001/jamanetworkopen.2024.60558 (PMC11840648; doi:10.1001/jamanetworkopen.2024.60558)
Supplement: Supplement 2. — Data Sharing Statement [file jamanetwopen-e2460558-s002.pdf]

Data Sharing Statement

Ukonaho. Socioeconomic Differences in Vaccination Coverage After a Mandatory Vaccination Law, 1855-1900. *JAMA Netw Open*. Published February 19, 2025.
doi:10.1001/jamanetworkopen.2024.60558

Data
Data available: Yes
Data types: Data dictionary
How to access data: The data that support the findings of this study are available on request from the corresponding author, SU (susanna.ukonaho@gmail.com)
When available: With publication

Supporting Documents
Document types: None

Additional Information
Who can access the data: The data will be available to anyone requesting the data.
Types of analyses: The data will be available for any purpose.
Mechanisms of data availability: The data will be available without investigator support.
